# Supplementary material for: Prevalence, incidence and predictors of cardiovascular risk factors: longitudinal data from rural and urban South India and comparison with global data
Source: BMJ Open Diabetes Res Care. 2020 Oct 22;8(1):e001782. doi: 10.1136/bmjdrc-2020-001782 (PMC7583064; doi:10.1136/bmjdrc-2020-001782)

**Supplementary Table 1.** Anthropometry, life style and cardiovascular risk factors (continuous variables) at Phase-5 (baseline) and Phase-6 (follow-up) of the Vellore Birth Cohort participants according to their place of residence (rural or urban)

| | Phase | Men | | Women | |
|---|---|---|---|---|---|
| | | Rural (n=240) | Urban (n=279) | Rural (n=217) | Urban (n=226) |
| Age, years, mean (SD) | Phase-5 | 28.0 (1.0) | 28.1 (1.1) | 28.3 (1.0) | 28.5 (1.1) |
| | Phase-6 | 42.0 (0.9) | 41.6 (1.0) | 42.1 (1.0) | 41.7 (1.0) |
| | % Change | 14.1 (0.7) | 13.9 (0.8) | 13.9 (0.6) | 13.2 (0.6) |
| Tobacco use, Yes, n (%) | Phase-5 | 77 (32.1) | 121 (43.4) | 0 (0.0) | 0 (0.0) |
| | Phase-6 | 53 (22.1) | 115 (41.2) | 0 (0.0) | 0 (0.0) |
| | Never | 157 (65.4) | 142 (50.9) | 217 (100) | 226 (100) |
| | Started | 6 (2.5) | 16 (5.7) | 0 (0.0) | 0 (0.0) |
| | Continued | 47 (19.5) | 99 (35.4) | 0 (0.0) | 0 (0.0) |
| | Stopped | 30 (12.5) | 22 (7.9) | 0 (0.0) | 0 (0.0) |
| Alcohol use, Yes, n (%) | Phase-5 | 101 (42.1) | 128 (45.9) | 0 (0.0) | 0 (0.0) |
| | Phase-6 | 132 (55.0) | 130 (46.6) | 0 (0.0) | 0 (0.0) |
| | Never | 92 (38.3) | 120 (43.0) | 217 (100) | 226 (100) |
| | Started | 47 (19.5) | 31 (11.1) | 0 (0.0) | 0 (0.0) |
| | Continued | 85 (35.4) | 99 (35.4) | 0 (0.0) | 0 (0.0) |
| | Stopped | 16 (6.6) | 29 (10.4) | 0 (0.0) | 0 (0.0) |
| SES, mean (SD) (Number of 5 Possessions) | Phase-5 | 2.0 (1.4) | 2.9 (1.1) | 1.7 (1.3) | 2.7 (1.1) |
| | Phase-6 | 3.3 (0.8) | 3.3 (0.8) | 3.2 (0.8) | 3.2 (0.9) |
| | % Change | 1.3 (1.3) | 0.4 (1.0) | 1.5 (1.2) | 0.5 (1.2) |
| Physical Activity Score, median (IQR) | Phase-5 | 1478 (819, 1905) | 1212 (743, 1747) | 1927 (1510, 2553) | 1719 (1322, 2202) |
| | Phase-6 | 1290 (838, 1441) | 1029 (780, 1416) | 1563 (1297, 1969) | 1555 (1184, 1942) |
| | % Change | -271 (-708, 213) | -61 (-502, 337) | -302 (-960, 68) | -212 (-608, 165) |
| Height, cm, mean, (SD) | | | | | |
| | Phase-5 | 165.1 (6.4) | 167.4 (7.2) | 153.7 (5.7) | 153.7 (5.4) |
| | Phase-6 | 164.7 (6.4) | 166.7 (7.3) | 153.6 (5.8) | 153.2 (5.3) |



Vasan SK, *et al. BMJ Open Diab Res Care* 2020; 8:e001782. doi: 10.1136/bmjdrc-2020-001782

| | | | | | |
|---|---|---|---|---|---|
| | % Change | -0.5 (1.3) | -0.7 (1.4) | -0.2 (2.1) | -0.5 (1.8) |
| Body mass index, | Phase-5 | 19.9 (3.0) | 21.5 (3.6) | 20.0 (3.5) | 23.1 (4.5) |
| kg/m$^2$, mean | Phase-6 | 23.5 (3.9) | 25.2 (4.4) | 25.0 (4.9) | 27.8 (5.2) |
| (SD) | % Change | 3.6 (2.7) | 3.6 (2.8) | 5.1 (3.3) | 4.8 (3.0) |
| Waist | Phase-5 | 74.9 (9.1) | 80.2 (11.4) | 69.4 (8.5) | 75.7 (10.3) |
| Circumference, cm, | Phase-6 | 86.8 (11.1) | 92.3 (12.3) | 81.1 (11.9) | 87.9 (11.7) |
| mean (SD) | % Change | 11.9 (7.4) | 12.2 (8.1) | 11.7 (9.0) | 12.2 (9.0) |
| Systolic blood | Phase-5 | 110.3 (10.8) | 114.0 (11.0) | 101.3 (10.4) | 101.4 (9.7) |
| pressure, mm Hg, | Phase-6 | 126.8 (15.1) | 127.5 (15.7) | 118.0 (13.8) | 118.6 (13.7) |
| mean (SD) | % Change | 16.4 (14.2) | 12.7 (16.2) | 16.7 (12.2) | 17.2 (11.5) |
| Diastolic blood | Phase-5 | 70.5 (8.7) | 74.7 (9.3) | 71.6 (8.3) | 73.3 (7.8) |
| pressure, mm Hg, | Phase-6 | 80.2 (12.2) | 79.4 (12.2) | 75.9 (10.0) | 76.2 (10.0) |
| mean (SD) | % Change | 9.7 (11.4) | 4.7 (12.5) | 4.3 (9.5) | 2.9 (9.3) |
| Fasting plasma | Phase-5 | 5.4 (5.1, 5.7) | 5.5 (5.2, 5.8) | 5.3 (4.9, 5.7) | 5.4 (5.1, 5.8) |
| glucose, mmol/L, | Phase-6 | 5.2 (4.8, 5.8) | 5.3 (4.9, 5.8) | 5.2 (4.8, 5.6) | 5.6 (4.9, 6.2) |
| median (IQR) | % Change | -0.11 (-0.56, 0.39) | -0.11 (-0.56, 0.44) | -0.06 (-0.44, 0.39) | 0.03 (-0.39, 0.67) |
| 120-min glucose, | Phase-5 | 5.9 (5.2, 7.1) | 6.4 (5.3, 7.4) | 6.4 (5.6, 7.3) | 6.7 (5.8, 7.7) |
| mmol/L, | Phase-6 | 6.2 (5.0, 8.5) | 6.2 (5.1, 8.2) | 6.4 (5.6, 7.8) | 6.8 (5.7, 8.4) |
| median (IQR) | % Change | 0.4 (-1.1, 2.4) | 0.1 (-1.4, 1.8) | 0.3 (-0.7, 1.8) | 0.1 (-0.8, 1.5) |
| Total cholesterol, | Phase-5 | 3.7 (0.8) | 4.3 (1.0) | 3.8 (0.7) | 4.2 (0.9) |
| mmol/L, | Phase-6 | 4.5 (1.0) | 4.8 (0.9) | 4.3 (0.8) | 4.6 (0.9) |
| mean (SD) | % Change | 0.7 (0.9) | 0.4 (1.0) | 0.5 (0.7) | 0.4 (0.9) |
| HDL cholesterol, | Phase-5 | 0.98 (0.25) | 0.99 (0.22) | 1.08 (0.24) | 1.09 (0.22) |
| mmol /L, | Phase-6 | 1.10 (0.29) | 1.04 (0.26) | 1.13 (0.22) | 1.18 (0.27) |
| mean (SD) | % Change | 0.12 (0.30) | 0.05 (0.26) | 0.06 (0.27) | 0.09 (0.26) |
| LDL cholesterol, | Phase-5 | 2.22 (0.66) | 2.74 (0.72) | 2.27 (0.62) | 2.65 (0.80) |
| mmol/L, | Phase-6 | 2.80 (0.76) | 3.14 (0.80) | 2.73 (0.66) | 3.00 (0.75) |
| mean (SD) | % Change | 0.58 (0.79) | 0.40 (0.74) | 0.45 (0.62) | 0.34 (0.79) |
| Triglycerides, | Phase-5 | 1.0 (0.7, 1.3) | 1.2 (0.8, 1.7) | 0.8 (0.6, 1.1) | 0.8 (0.6, 1.2) |
| mmol/L, | Phase-6 | 1.4 (1.0, 2.1) | 1.5 (1.0, 2.3) | 1.0 (0.8, 1.5) | 1.0 (0.8, 1.5) |
| median (IQR) | % Change | 0.4 (0.0, 1.0) | 0.3 (-0.1, 0.9) | 0.2 (0.0, 0.6) | 0.2 (-0.1, 0.5) |

SD – standard deviation; IQR – inter-quartile range

**Supplementary Table 2.** Anthropometry and life style factors in Phase-5: comparison between cohort members studied and not studied in Phase-6

| Measurement | | Men | | Women | |
|---|---|---|---|---|---|
| | | Rural | Urban | Rural | Urban |
| n | Not Studied | 377 | 265 | 387 | 227 |
| | Studied | 240 | 279 | 217 | 226 |
| Age, years, mean | Not Studied | 27.9 (1.1) | 28.3 (1.2) | 28.2 (1.5) | 28.5 (1.1) |
| (SD) | Studied | 28.0 (1.0) | 27.7 (1.2) | 28.1 (1.0) | 28.6 (1.1) |
| | P value | 0.815 | <0.001 | 0.958 | 0.656 |
| Tobacco use | Not Studied | 119 (31.2) | 109 (41.1) | 0 (0) | 0 (0) |
| Yes, n (%) | Studied | 77 (32.1) | 121 (43.4) | 0 (0) | 0 (0) |
| | P value | 0.893 | 0.598 | 0 (0) | 0 (0) |
| Alcohol use | Not Studied | 160 (42.4) | 146 (55.1) | 0 (0) | 1 (0.4) |
| Yes, n (%) | Studied | 101 (42.1) | 128 (45.9) | 0 (0) | 0 (0) |
| | P value | 0.930 | 0.032 | 0 (0) | 0 (0) |
| SES, mean (SD), | Not Studied | 2.0 (1.3) | 2.7 (1.2) | 1.9 (1.4) | 2.5 (1.2) |
| (Number of 5 | Studied | 2.0 (1.4) | 2.9 (1.1) | 1.7 (1.3) | 2.7 (1.1) |
| Possessions) | P value | 0.652 | 0.054 | 0.274 | 0.077 |
| Physical Activity | Not Studied | 1469 | 1438 | 1938 | 1747 |
| Score, | | (983, 5743) | (871, 1815) | (1487, 2552) | (1348, 2262) |
| median (IQR) | Studied | 1478 | 1212 | 1928 | 1719 |
| | | (819, 1905) | (743, 1748) | (1510, 2553) | (1323, 2202) |
| | P value | 0.394 | 0.001 | 0.993 | 0.306 |
| Height, cm, mean | Not Studied | 166.1 (6.1) | 167.3 (6.5) | 153.7 (6.2) | 153.4 (5.5) |
| (SD) | Studied | 165.1 (6.4) | 167.4 (7.2) | 153.7 (5.7) | 153.7 (5.4) |
| | P value | 0.049 | 0.979 | 0.972 | 0.611 |
| Body mass index, | Not Studied | 20.0 (3.3) | 21.4 (3.5) | 19.6 (3.7) | 21.5 (4.0) |
| ($kg/m^2$) | Studied | 19.9 (3.0) | 21.5 (3.6) | 20.0 (3.5) | 23.1 (4.5) |
| mean (SD) | P value | 0.927 | 0.706 | 0.212 | <0.001 |
| Waist | Not Studied | 76.3 (10.1) | 79.3 (11.0) | 68.4 (8.8) | 72.1 (8.9) |
| Circumference, | Studied | 74.9 (9.1) | 80.2 (11.4) | 69.4 (8.5) | 75.7 (10.3) |
| cm, mean (SD) | P value | 0.077 | 0.348 | 0.171 | <0.001 |

SD – standard deviation; IQR – inter-quartile range. [a]p-values from independent-t-test; [b]p-values from chi-square statistics; [c]p-values from t-test after log transformation

**Supplementary Table 3.** Cardiovascular risk factors in Phase-5: comparison between cohort members studied and not studied in Phase-6

| Measurement | | Men | | Women | |
|---|---|---|---|---|---|
| | | Rural | Urban | Rural | Urban |
| n | Not Studied | 377 | 265 | 387 | 227 |
| | Studied | 240 | 279 | 217 | 226 |
| Systolic blood | Not Studied | 110.9 (11.0) | 113.6 (10.8) | 101.8 (11.2) | 100.8 (10.8) |
| pressure, mm Hg, | Studied | 110.3 (10.8) | 114.8 (11.0) | 101.3 (10.4) | 101.4 (9.7) |
| mean, (SD) | P value[a] | 0.498 | 0.198 | 0.566 | 0.537 |
| Diastolic blood | Not Studied | 71.3 (9.3) | 74.9 (9.1) | 72.7 (8.7) | 72.5 (8.5) |
| pressure, mm Hg, | Studied | 70.5 (8.7) | 74.7 (9.3) | 71.6 (8.3) | 73.3 (7.8) |
| mean, (SD) | P value[a] | 0.254 | 0.838 | 0.142 | 0.301 |
| Fasting plasma | Not Studied | 5.4 (5.1, 5.7) | 5.4 (5.0, 5.8) | 5.3 (5.0, 5.6) | 5.3 (5.0, 5.7) |
| glucose, mmol/L, | Studied | 5.4 (5.1, 5.7) | 5.5 (5.2, 5.8) | 5.3 (4.9, 5.7) | 5.4 (5.1, 5.8) |
| median (IQR) | P value[b] | 0.138 | 0.181 | 0.932 | 0.030 |
| 120-min glucose, | Not Studied | 5.9 (5.1, 6.9) | 6.3 (5.3, 7.4) | 6.2 (5.3, 7.1) | 6.5 (5.6, 7.7) |
| mmol/L, | Studied | 5.9 (5.2, 7.1) | 6.4 (5.3, 7.4) | 6.4 (5.6, 7.3) | 6.7 (5.8, 7.7) |
| median (IQR) | P value[b] | 0.600 | 0.762 | 0.870 | 0.261 |
| Total cholesterol, | Not Studied | 3.8 (0.9) | 4.3 (0.9) | 3.8 (0.8) | 4.0 (0.8) |
| mmol/L, | Studied | 3.7 (0.8) | 4.3 (1.0) | 3.8 (0.7) | 4.2 (0.9) |
| mean (SD) | P value[a] | 0.073 | 0.630 | 0.997 | 0.007 |
| HDL cholesterol, | Not Studied | 0.97 (0.23) | 0.97 (0.24) | 1.10 (0.23) | 1.08 (0.25) |
| mmol/L, | Studied | 0.98 (0.25) | 0.99 (0.22) | 1.08 (0.24) | 1.09 (0.22) |
| mean (SD) | P value[a] | 0.498 | 0.270 | 0.328 | 0.500 |
| LDL cholesterol, | Not Studied | 2.35 (0.72) | 2.70 (0.76) | 2.28 (0.69) | 2.47 (0.65) |
| mmol/L, | Studied | 2.22 (0.66) | 2.74 (0.72) | 2.27 (0.62) | 2.65 (0.80) |
| mean (SD) | P value[a] | 0.027 | 0.513 | 0.852 | 0.009 |
| Triglycerides, | Not Studied | 0.98 | 1.10 | 0.78 | 0.80 |
| mmol/L, | | (0.70, 1.34) | (0.82, 1.66) | (0.58, 1.00) | (0.63, 1.08) |
| median (IQR) | Studied | 0.95 | 1.17 | 0.75 | 0.83 |

| | | | | |
|---|---|---|---|---|
| | (0.72, 1.31) | (0.81, 1.70) | (0.56, 1.08) | (0.62, 1.17) |
| P value[b] | 0.616 | 0.674 | 0.493 | 0.512 |

[a]p-values from independent-t-test; [b]p-values from t-test after log transformation. SD – standard deviation; IQR – inter-quartile range

**Supplementary Figure 1**. Kernel density plots showing the change in total-, HDL- and LDL-cholesterol from Phase-5 to Phase-6 in rural and urban men and women.

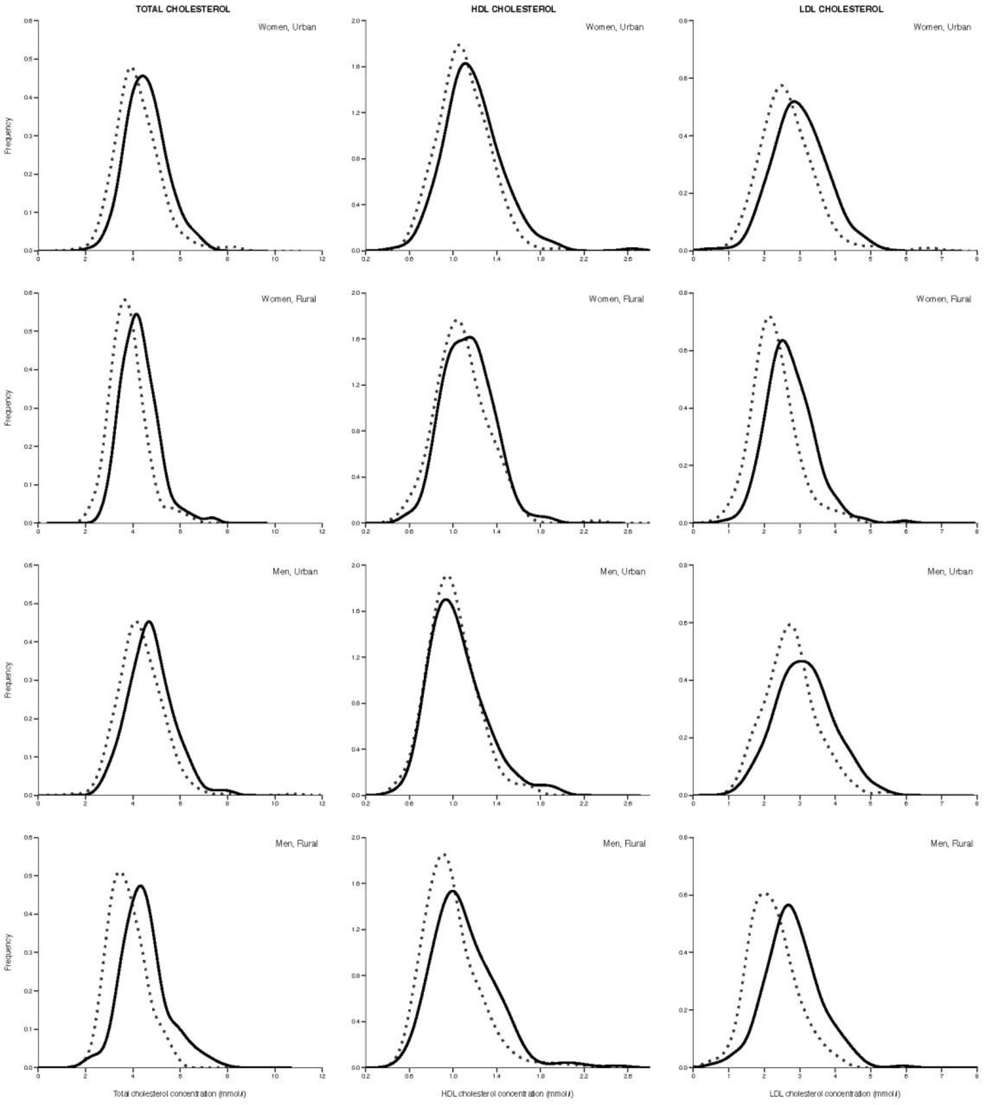

Supplement: Supplementary data [file bmjdrc-2020-001782supp001.pdf]
